# Discriminating Marine Macroalgae by Volatilomic Fingerprint and Bioactivity: A Chemometric Approach

**DOI:** 10.3390/biology15141129

**Published:** 2026-07-11

**Authors:** Gonçalo Jasmins, Rosa Perestrelo, Ricardo Luís, Rodrigo Silva, Pedro Sousa, Carlos A. P. Andrade, José Câmara

**Affiliations:** 1 CQM—Centro de Química da Madeira, Universidade da Madeira, Campus da Penteada, 9020-105 Funchal, Portugal; goncalo.jasmins@staff.uma.pt (G.J.); rmp@staff.uma.pt (R.P.); 2 MARE—Marine and Environmental Sciences Centre/ARNET—Aquatic Research Network, Agência Regional Para o Desenvolvimento da Investigação Tecnologia e Inovação (ARDITI), 9020-105 Funchal, Portugal; ricardo.luis@arditi.pt (R.L.); rodrigo.silva@mare.arditi.pt (R.S.); pedro.sousa@mare.arditi.pt (P.S.); 3 Departamento de Química, Faculdade de Ciências Exatas e Engenharia, Universidade da Madeira, Campus da Penteada, 9020-105 Funchal, Portugal

**Keywords:** marine macroalgae, volatilomic fingerprint, antioxidant capacity, HS-SPME/GC-MS, *in chemico* assays, multivariate analysis

## Abstract

Marine macroalgae are now considered potential sources of renewable feedstocks to produce valuable bioactive compounds. However, the species-specific volatilomes and their correlation with antioxidant capacity remains largely unknown. This study aimed to investigate the volatilomic fingerprint, total phenolic content (TPC), total flavonoids content (TFC) and antioxidant capacity of four macroalgae belonging to distinct taxonomic groups: *Rugulopteryx okamurae* (brown algae), *Asparagopsis taxiformis* (red algae), *Caulerpa webbiana* (green algae), and coralline algae. To achieve this purpose, the volatilomic fingerprint was established using headspace solid-phase microextraction tandem gas chromatography–mass spectrometry (HS-SPME/GC-MS), and the TPC, TFC, and antioxidant capacity were evaluated using *in chemico* assays. A total of 59 volatile organic compounds (VOCs) were identified across the marine macroalgae, encompassing carbonyl compounds (on average, 40.7 ± 13.4% of total volatilomic fraction), hydrocarbons (17.0 ± 8.3%), organohalogens (23.0 ± 10.8%), terpenoids (6.5 ± 2.0%), alcohols (4.5 ± 2.0%), and the remaining chemical families showed a contribution lower than 5% to the total volatilomic fingerprint. Multivariate statistical analyses revealed significant differences (*p* < 0.05) in the volatilomic fingerprint among the macroalgae investigated, allowing clear discrimination between species. Organohalogen compounds emerged as key discriminant features for *C. webbiana* and *A. taxiformis* algae, while hydrocarbons were characteristic of the *R. okamurae*. Despite taxonomic differences, hexanal, benzaldehyde and β-ionone were VOCs found across all samples. Regarding bioactivity, *R. okamurae* exhibited the highest TPC (144 mgGAE/100 g DW), TFC (69 ± 6 mg QE/100 g DW), and antioxidant capacity. These findings confirm the large variability of the volatilomes of the different species of macroalgae and demonstrate the power of the HS-SPME/GC-MS, chemometric, and antioxidant analysis strategy for the differentiation of species and the identification of VOCs with valorization potential, helping to transform invasive or unused biomass of these species into new, sustainable bioproducts through emerging technological and biorefinery applications.

## Simple Summary

Marine macroalgae have attracted increasing interest as renewable resources to produce high-value bioactive compounds with applications in the food, pharmaceutical and cosmetic industries. Nevertheless, the volatile composition and antioxidant properties of many species remain under-characterized. This study investigated the volatile organic compound profiles, phenolic and flavonoid contents, and antioxidant capacity of four macroalgae belonging to different taxonomic groups: *Rugulopteryx okamurae*, *Asparagopsis taxiformis*, *Caulerpa webbiana*, and crustose coralline algae. Each alga exhibited a distinct volatilomic fingerprint, allowing a clear differentiation among species. Within the species investigated, *R. okamurae* displayed the highest antioxidant potential, as well as the highest total flavonoid and phenolic content. These findings expand on current knowledge of the chemical diversity of marine macroalgae and demonstrate their potential as sustainable sources of valuable natural compounds, supporting the valorization of invasive and underutilized biomass within a circular bioeconomy.

## 1. Introduction

Algae comprise a wide range of organisms found across multiple habitats, including ponds, lakes, rivers, oceans [1]. Algae exhibit extensive morphological and physiological diversity, ranging from unicellular microscopic forms to large multicellular macroalgae, such as kelps, which can reach lengths of up to 60 m. Through oxygenic photosynthesis, algae significantly contributed to the oxygenation of the early Earth’s atmosphere, thereby enabling the emergence of aerobic metabolism and the subsequent development of habitable environmental conditions [2]. Beyond their ecological importance, macroalgae represent a valuable natural resource due to their rich and diverse chemical composition which includes fatty acids, carotenoids, vitamins, minerals, dietary fibers, volatile organic compounds (VOCs) and proteins, among other bioactive metabolites [3]. These compounds have attracted an increasing interest both in the scientific and industrial sectors due to their potential applications; however, algae are often misunderstood and undervalued by the public, despite supporting a multi-billion-dollar industry [2]. In recent years, the transition towards a more sustainable and resource-efficient bioeconomy has intensified the search for renewable marine resources capable of supporting economic development while minimizing environmental impact. Consistent with United Nations directives, countries are expected to formulate and implement targeted strategies aimed at achieving a renewable society. However, the effective utilization of marine resources, coupled with the need to reconcile economic development with environmental protection, remains a critical challenge [1].

Macroalgae are broadly classified into three major taxa—green (*Chlorophyta*), red (*Rhodophyta*), and brown (*Heterokontophyta*)—based on their characteristic pigmentation [1]. Green algae comprise a highly diverse and structurally complex group of organisms distributed across most photic habitats, characterized by the presence of chlorophylls *a* and *b*, which confers their characteristic green pigmentation [4]. Green algae exhibit species-dependent variability, for example *Caulerpa* sp. are rich in terpenoids and alkaloids [5], while *Ulva* sp. are rich in ulvans (sulphated polysaccharides) [6]. A previous headspace solid-phase microextraction combined with gas chromatography–mass spectrometry (HS-SPME/GC-MS) approach established the volatilomic fingerprint of *Caulerpa* species; nonanoic acid, octanol, dendrolasin, tridecanal, nonanal, (*E*,*E*)-2,4-dodecadienal, (*E*,*E*)-2,4-heptadienal, and (*E*)-2-octenal identified as key VOCs of these species [6].

Red algae’s characteristic color is manifested by the presence of phycoerythrins [7]. They possess a complex biochemical composition, including polysaccharides such as agar and carrageenans, which have been shown to exhibit antidiabetic and antitumor activities. These polysaccharides not only serve as rich sources of soluble dietary fibers but also play a critical role in inhibiting key enzymes linked to carbohydrate metabolism and glucose uptake, thereby offering promising therapeutic potential in managing diabetes. Furthermore, these algae are also rich in polyphenols, flavonoids, and lectins, contributing to their bioactive versatility. The chemical composition and, consequently, the volatilomic fingerprint of *A. taxiformis* are likely to vary with the time and location of collection, hindering the establishment of a consistent volatile profile due to the limited data available [8].

Red algae, as well as green algae, are known to metabolize halogenated VOCs primarily from chlorine and/or bromine. These VOCs play key roles in the algae’s defense systems, aiding in growth, reproduction, and protection against predators and pathogens. The red algae, particularly the genus *Laurencia*, are among the most prevalent producers of halogenated compounds, many of which exhibit antibacterial, antifungal, antiviral and cytotoxic properties [9].

Within red algae, crustose coralline algae (pinkish calcifying red algae), represent a specialized calcifying group characterized by the deposition of calcium carbonate within their cell walls, which confers a rigid structure and enables them to play a key role in marine ecosystems by stabilizing substrates and providing habitat for diverse organisms [10]. These marine algae contain high levels of eicosapentaenoic acid, an omega-3 fatty acid, as well as carotenoids (α-carotene and β-carotene), which are known to have antioxidant and anticancer activities [11]. These algae are also known to be important contributors to the marine sulfur cycling, through the production of Dimethylsulphoniopropionate (DMSP), which can subsequently be degraded into dimethyl sulphide (DMS). DMS is a volatile sulphur compound that, upon reaching the atmosphere, it oxidizes and forms aerosols. These sulphur-containing aerosols may contribute to the cloud condensation nuclei formation, thus influencing Earth’s climate regulation. [12]. Even though their bioactive compounds and ecological importance are immense, their volatilomic signature remains comparatively underexplored.

Brown algae constitute another highly diverse group, displaying morphological complexity and pigments dominated by the xanthophyll fucoxanthin. They are rich in bioactive compounds such as phlorotannins, fucoxanthin, alginic acid and fucoidan, which have been associated with antioxidants, anti-inflammatory and other health-related properties [13]. As for the volatilomic signature, brown algae have been reported to be predominantly characterized by pentadecane [14]. The invasive species *R. okamurae*, which originated from the Pacific Ocean, has spread extensively along the European coastlines (including Madeira Island), causing massive environmental and ecological issues. This species outcompetes the native ones by having periods of massive biomass generation, interfering with the natural habitat, and afterwards its proliferation declines and deposits on the shores causing huge challenges for coastal management [15]. Therefore, the search for its bioactive potential as a food and/or cosmetic source has also increased to offset the overabundance of this species.

The aim of this study was to characterize and compare the volatilomic fingerprints of four macroalgal species—*Caulerpa webbiana* (green algae), *Asparagopsis taxiformis* and crustose coralline algae (red algae) and *Rugulopteryx okamurae* (brown algae)—using HS-SPME/GC–MS. Discriminant volatile organic compounds (VOCs) were identified through multivariate statistical analyses. The bioactive potential was evaluated by determining total phenolic content (TPC), total flavonoid content (TFC), and antioxidant capacity via *in chemico* assays. In addition, the potential valorisation of these species as sustainable sources of bioactive compounds for food and cosmetic applications was explored. Finally, this study aimed to evaluate the robustness and suitability of HS-SPME/GC-MS combined with multivariate statistical analyses as a framework for the characterization and differentiation of marine macroalgae volatilomic profile.

## 2. Materials and Methods

### 2.1. Chemicals and Reagents

All chemicals and reagents used were of analytical grade. Sodium chloride (NaCl, 99.5%), 3-octanol (internal standard (IS), 99%) and 2,2′-azino-bis(3-ethylbenzothiazoline-6-sulfonic acid) radical cation (ABTS, 98%) were purchased from Sigma-Aldrich (St. Louis, MO, USA). The SPME fiber coated with divinylbenzene/carboxen/polydimethylsiloxane (DVB/CAR/PDMS) (50/30 μm), SPME holder for manual sampling, and glass vials were purchased from Supelco (Bellefonte, PA, USA). Ultrapure water (18 MΩ cm) was provided by the Milli-Q water purification system (Millipore, Milford, MA, USA). The alkane series (C8 to C20, 40 mg/L in n-hexane), Folin–Ciocalteu reagent (FR, 2 N), 2,2-diphenyl-1-picrylhydrazyl (DPPH^•^), 6-hydroxy-2,5,7,8-tetramethylchromane-2-carboxylic acid (trolox, 98%) and gallic acid (C_7_H_6_O_5_, 98%) were supplied by Fluka (Buchs, Switzerland). Methanol was purchased by Fischer Scientific (Loughborough, UK). Quercetin (99%) was supplied by Acros Organics (Geel, Belgium). Aluminum chloride (AlCl_3_) and potassium chloride (KCl, >99%) were acquired by Riedel-de Haën^®^ (Seelze, Germany). Sodium carbonate (Na_2_CO_3_, >99%) was obtained by Labsolve^®^ (Lisboa, Portugal), while potassium persulfate (K_2_S_2_O_8_) was purchased from Merck^®^ (Darmstadt, Germany).

### 2.2. Sample Collection and Preparation

**Figure 1:**
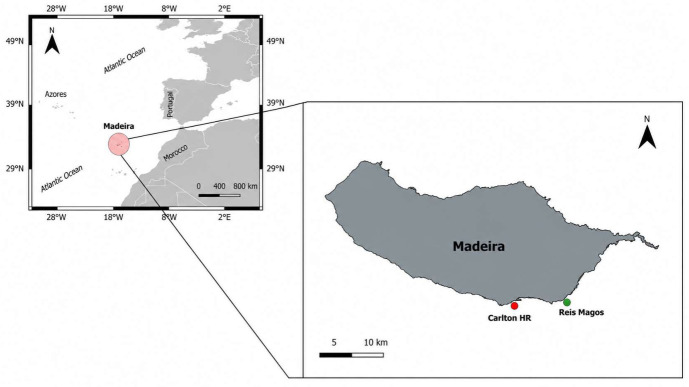
Sites of collection of marine macroalgae.

Rocks containing crustose coralline algae (CCA) were collected at Praia dos Reis Magos (Santa Cruz, Madeira Island, 32.647343, −16.823683) (Figure 1), and transported to Centro de Maricultura da Calheta (Calheta, Madeira Island, Portugal).

**Figure 2:**
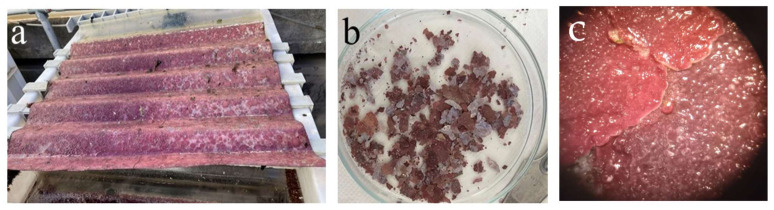
(**a**) Coralline algae encrusted on the PVC plate; (**b**) coralline algae removed from the PVC plate; (**c**) coralline algae under optic microscopy view.

Upon arrival, they were sorted according to macroscopic color morphotype and distributed into two 200 L, flat-bottom PVC rectangular tanks with artificial aeration and an open flow-through system (water flow of approximately 90 L per hour) (Figure 2a). Each contained eight corrugated, transparent PVC plates aimed to promote algal attachment and colonization. The tanks operated under fully natural environmental conditions, including ambient seawater temperature and unaltered light and flow regimes. Coralline algae species identification could not be achieved by its macroscopic morphology alone.

Whole thalli of *C. webbiana*, *A. taxiformis* and *R. okamurae* samples were collected in June 2025 at Madeira DivePoint, Hotel Carlton House reef (Funchal, Madeira Island, coordinates: 32.641161, −16.921525) (Figure 1), by three divers at depths ranging from 0 to 10 m. The collection method entailed in situ harvesting, where entire algal individuals, including all visible vegetative structures were sampled and processed for subsequent analyses. Only fully developed individuals displaying the diagnostic morphological characteristics described for adult thalli of each species were selected. After collection, the algae were immediately stored in zip bags containing seawater. At the laboratory, the algae were thoroughly rinsed with tap water and subsequently with distilled water to eliminate any epiphytes and residual debris and stored at −20 °C (in 50 mL amber vials).

### 2.3. Extraction of the Bioactive Compounds for the in Chemico Assays

The algae utilized for the *in chemico* assays were lyophilized (Telstar, Cryodos, Barcelona, Spain) for 72 h, then milled using a laboratory mill (IKA^®^ A11 basic, Guangzhou, China) to obtain a fine-like powder and stored in 50 mL Falcon tubes at −20 °C until further use.

The extraction of bioactive compounds from marine macroalgae was conducted by adding 1 g of algae powder and 25 mL of methanol:H_2_O (1:1 *v*/*v* %) into a 50 mL Falcon tube and letting it stir for one hour. Afterwards, the Falcon tubes were taken to the ultrasound bath (Branson 2510, Markham, ON, Canada) for 10 min and finally centrifuged (Sigma 1–6p) for 10 min at 3000 rpm. The supernatant was then stored in a different Falcon tube at −20 °C until analysis [16].

### 2.4. In Chemico Assays to Assess the Phenolic Composition and Antioxidant Capacity

*In chemico* assays were applied to evaluate the phenolic composition and antioxidant capacity of the marine macroalgae. All the spectrophotometric measurements were done in triplicate using a UV-Vis spectrophotometric (Lambda 25, PerkinElmer, Waltham, MA, USA).

The total phenolic content (TPC) assay was determined by the Folin–Ciocalteu assay, and the total flavonoid content (TFC) was determined by the AlCl_3_ colorimetric assay described by Abreu et al. [17]. The TPC protocol was performed by adding 0.6 mL of the extract, plus 3 mL of Folin–Ciocalteu (1:10) and 2.4 mL of Na_2_CO_3_ 7.5% (*w*/*v*) onto a 15 mL Falcon tube. Thereafter, the tube was vortexed and allowed to stand for 30 min. The absorbance was measured at 765 nm. The results of the TPC were expressed in milligrams of gallic acid equivalent per 100 g of dry sample (mgGAE/100 g DW), considering that gallic acid was used as the standard to plot the calibration curve with concentrations ranging from 50 to 175 mg/L.

The TFC protocol was carried out by adding 3 mL of the extract and 3 mL of 2% AlCl_3_ (*w*/*v*) into the Falcon tube. The samples were kept to rest for 10 min before measuring their absorbance at 440 nm. The TFC assay data were expressed as milligrams of quercetin equivalent per 100 g of dry sample (mgQE/100 g DW), based on a calibration curve constructed with quercetin concentrations ranging from 5 to 30 mg/L.

The antioxidant capacity was assessed using DPPH free radical-scavenging activity and ABTS antioxidant activity methods described by Abreu et al. [17]. In short, a stock solution of DPPH radical was prepared in methanol and maintained in the dark at 25 °C. The stock solution was diluted in methanol to obtain a 0.900 (±0.030) absorbance working solution at 515 nm. Then, 3.9 mL of the DPPH solution was added onto a 15 mL Falcon tube, followed by 100 µL of algae extract (tube was vortexed for 15 s) and was kept in a light absent place for 45 min. Trolox was utilized as the standard to construct the calibration curve with concentrations ranging from 25 to 450 mg/L. ABTS^+^ radical cations were generated by reacting ABTS (20 mM) with K_2_S_2_O_8_ (70 mM) and incubating in the dark for 16 h. This solution was diluted in PBS to obtain an absorbance of 0.900 (±0.030) working solution at 734 nm. After that, 3 mL of ABTS and 12 μL of the algae extract were added to a 15 mL Falcon tube and left in a dark place for 20 min. Trolox was also used as the standard to construct the calibration curve with concentrations ranging from 150 to 1000 mg/L. For both *in chemico* assays, the results were expressed in milligrams of trolox equivalent per 100 g of dry sample (mgTE/100 g DW).

### 2.5. Headspace-Solid Phase Microextraction

The SPME method used in this study was based on protocols previously described by Nor et al. [18], with a few alterations. The HS-SPME extraction consisted of adding 1 g of fresh algae cut into tiny pieces into a 20 mL amber vial, followed by the addition of 0.3 g of NaCl, 6 mL of H_2_O, 5 μL of 3-octanol (IS, 16.4 μg/L) and finally a magnetic stirring bar. The vial was then sealed with a PTFE-faced silicone septum and placed in a thermostatic bath. The DVB/CAR/PDMS fiber was inserted into the vial’s headspace to extract the VOCs for 50 min with a constant stirring of 300 rpm at 45 °C. Subsequently, the fiber was removed from the vial and inserted onto the GC injector port for 7 min at 250 °C. The samples were analyzed in triplicate with independent aliquots. Prior to each use, the SPME fiber was thermally conditioned at 270 °C for 30 min, according to the manufacturer’s instructions, to prevent the analytes carryover.

### 2.6. Gas Chromatography–Mass Spectrometry Conditions

The VOCs separation and identification were performed by an Agilent Technologies 6890 N Network gas chromatograph system (Palo Alto, CA, USA) equipped with a SUPELCOWAX 10 fused silica column (60 m × 0.25 mm I.D. × 0.25 μm film thickness, SGE, Dortmund, Germany) connected to an Agilent 5975 quadrupole inert mass selective detector. The injection port operated in splitless mode with a 250 °C stable temperature. The flow of the column was maintained at a constant 1.0 mL/min using helium (He, N60, Air Liquide, Algés, Portugal) as the carrier gas. The oven ramp started at 40 °C and maintained it for 3 min, increased 4 °C/min up till 220 °C for a total run time of 58 min. As for the MS detector, the operating temperatures of the transfer line, quadrupole and ionization source were 250, 150 and 230 °C, respectively. The electron impact mass spectra were recorded at 70 eV and the ionization current was 10 μA. The data acquisition was performed in scan mode from 30 to 300 *m*/*z*. The VOC identification was done by comparing the GC retention times (RT), Kovats index (KI) and mass spectra with those of the standard, when available, with a matching probability of 80%. The VOCs experimental Kovat index were supported by comparison with the literature values reported for the similar columns [19]. The National Institute of Standards and Technology (NIST) MS 05 spectral database (Gaithersburg, MD, USA) was also employed to compare with the mass spectra. The van den Dool and Kratz equation were used to determine the KI values [20], and then subsequently compared with the data reported in the literature for equivalent chromatographic columns. The C8–C20 n-alkanes series were analyzed under the same experimental conditions as the samples, to obtain the calculated KI for the identified VOCs and compare them with the KI available in the literature for equivalent columns. The identified VOCs were expressed as the mean relative area ± standard deviation. The relative peak area was calculated by the ratio between the GC peak areas of the VOC’s and the IS. The relative concentration of VOCs was estimated based on the added amount of 3-octanol (IS) and expressed as µg/L of 3-octanol equivalents.

### 2.7. Statistical Analysis

The statistical analysis was executed by a web-based platform designated as Metaboanalyst 6.0 [21]. Data normalization was performed by sum, transformation by log base 2 with auto scaling. After processing the data, it was subjected to one-way analysis of variance (ANOVA) and non-significant analytes were removed from the data set by employing Tukey’s post hoc test with a statistical significance of *p* < 0.05. Multivariate analysis was performed to detect discriminant VOCs through principal component analysis (PCA) and partial least squares discriminant analysis (PLS-DA). VOCs with a variable importance in projection (VIP) score higher than 1, were considered to be promising biomarkers to discern marine macroalgae samples. Hierarchical cluster analysis (HCA) was executed using VOCs with VIPs higher than 1, implementing the Euclidean distance and Ward’s linkage approach to identify clustering trends and associations between the algae. The model validation was performed using 5-fold cross-validation to appraise the classification performance. The PLS-DA model was also appraised through permutation testing (*n* = 1000).

## 3. Results and Discussion

### 3.1. Phenolic Composition and Antioxidant Capacity

A clear difference among the macroalgae is observed for both *in chemico* assays. One-way ANOVA showed significant differences between samples for both TFC (F(3,8) = 212.19, *p* < 0.001), TPC (F(3,8) = 235.95, *p* < 0.001) and antioxidant activities assessed by ABTS (F(3,8) = 36.59, *p* < 0.05) and DPPH (F(3,8) = 69.61, *p* < 0.05) assays.

**Figure 3:**
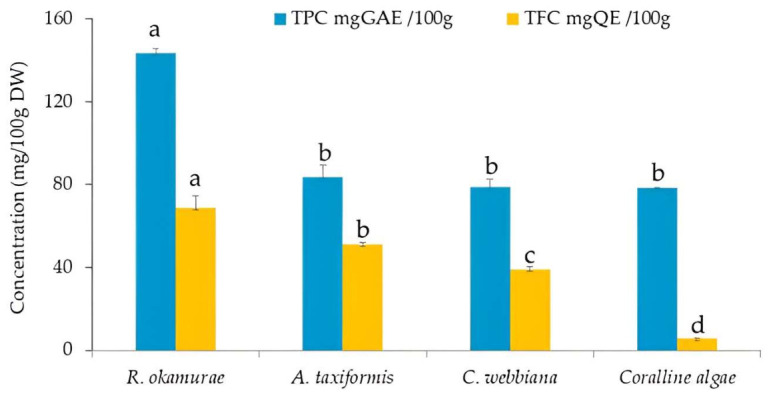
Total phenolic (mgGAE/100 g DW) and flavonoid (mgQE/100 g DW) content of marine macroalgae. Bars represent mean values ± standard deviation (*n* = 3). Different letters indicate statistically significant differences between samples according to one-way ANOVA followed by Tukey’s post hoc test (*p* < 0.05).

Regarding TFC, Tukey’s post hoc test indicated significant differences among all samples (Figure 3). *Rugulopteryx okamurae* (69 ± 6 mg QE/100 g) exhibited the highest content, followed by *Asparagopsis taxiformis* (51.1 ± 0.8 mg QE/100 g) and *Caulerpa webbiana* (39 ± 1 mg QE/100 g), whereas coralline algae showed markedly lower levels (5.66 ± 0.12 mg QE/100 g). With respect to TPC, again *R. okamurae* (144 ± 2 mgGAE/100 g) had significantly higher levels than the other samples. In contrast, no significant differences were observed among *A. taxiformis* (84 ± 6 mgGAE/100 g), *C. webbiana* (79 ± 4 mgGAE/100 g), and coralline algae (78.4 ± 0.1 mgGAE/100 g) samples (*p* > 0.05).

**Figure 4:**
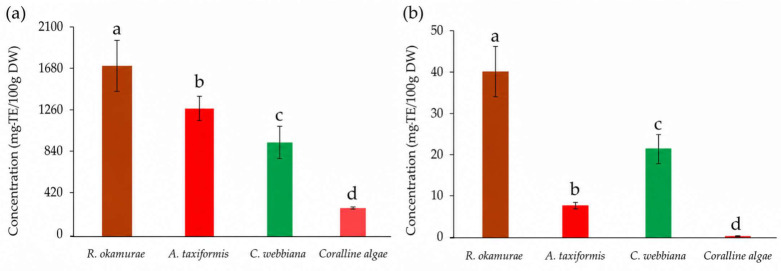
(**a**) ABTS and (**b**) DDPH radical scavenging activities of the marine macroalgae. Bars represent mean values ± standard deviation (*n* = 3). Different letters indicate statistically significant differences between samples according to one-way ANOVA followed by Tukey’s post hoc test (*p* < 0.05).

Antioxidant activity results of the different algae, as measured by the ABTS assay (Figure 4a), also exhibited a similar pattern to that of TPC and TFC. *R. okamurae* exhibited the highest antioxidant activity among the evaluated samples, as determined by the *in chemico* antioxidant assays, with a radical scavenging activity of 1784 ± 283 mgQE/100 g DW, followed by *A. taxiformis* (1311 ± 120 mgQE/100 g DW), *C. webbiana* (947 ± 180 mgQE/100 g DW), and coralline algae (310 ± 15 mgQE/100 g DW). All reported values differed significantly from each other, as confirmed by statistical analysis (*p* < 0.05), indicating the presence of statistically distinct groups among the samples.

In contrast, antioxidant activity, measured by the DPPH assay (Figure 4b), did not show similar trends to those observed for TPC and TFC. *R. okamurae* was again found to have the highest antioxidant activity, with a DPPH activity of 41 ± 6 mgQE/100 g DW. Conversely, coralline algae demonstrated minimal antioxidant activity, with values approaching to zero activity (0.31 ± 0.05 mgQE/100 g DW). *C. webbiana* demonstrated a significant increase in DPPH activity relative to the other tested samples (22 ± 4 mgQE/100 g DW) compared to *A. taxiformis* (8 ± 1 mgQE/100 g DW), although its TPC and TFC were lower than those of *A. taxiformis*. The established antioxidant capacity of the examined macroalgae, particularly of the invasive brown macroalgae *R. okamurae*, further emphasizes their potential in terms of being used in a circular economy strategy to transform a low-value or even nuisance material into a high-value functional ingredient via a sustainable extraction process.

The TPC, TFC and antioxidant capacity determined for the investigated marine macroalgae were comparable to those previously reported for *R. okamurae* (TPC = 310 ± 22 mgGAE/100 g DW; ABTS = 1489 ± 107 μmolTE/100 g DW; DPPH = 40.8 ± 0.5 mgAAE/100 g DW) [15], *A. taxiformis* (TPC = 57 ± 4 mgGAE/100 g DW; TFC = 19.3 ± 0.9 mgQE/100 g DW; DPPH = 21 ± 1 mgAAE/100 g DW) [16]. In contrast, the only study available for *C. webbiana* reported lower TPC, TFC, and antioxidant activity values than those obtained in the present work (TPC = 7.89 ± 0.94 mgGAE/g DW; TFC = 5.97 ± 0.22 mgGAE/g DW; DPPH = 2.37 ± 0.13 mgTE/g DW) [22]. Regarding the coralline algae, no comparable studies evaluating *in chemico* assays were found; the only available report concerned a geniculate coralline alga, limiting direct comparisons [23]. This quantitative difference may be attributed to different extraction procedures, solvent systems, sample-to-solvent ratios and *in chemico* antioxidant assay protocols, which strongly influence the recovery, composition and measured bioactivity of phenolic compounds.

### 3.2. Volatilomic Fingerprint of Marine Macroalgae

**Figure 5:**
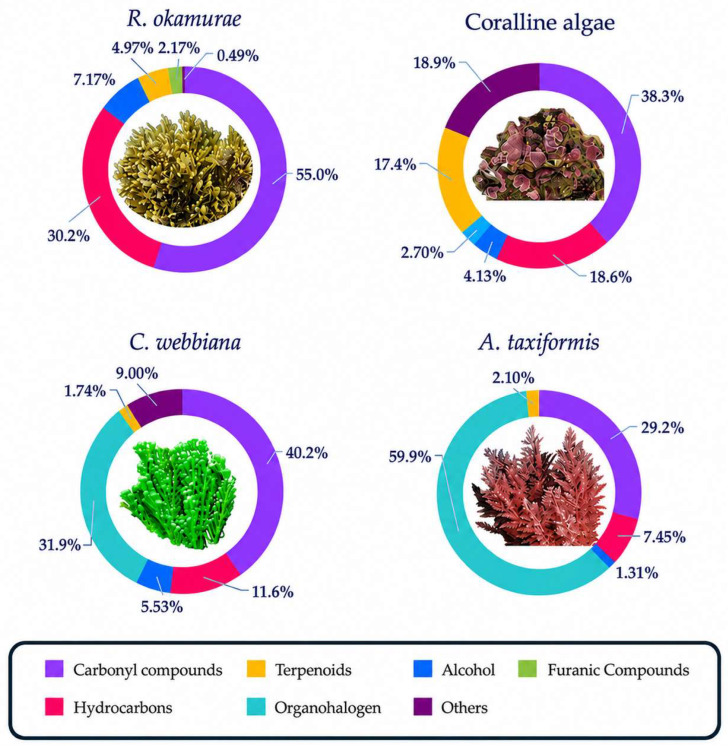
Contribution of each chemical family to the total volatilomic fingerprint of the marine macroalgae investigated.

A total of 59 VOCs were identified across the four algae samples using the HS-SPME/GC-MS approach (Supplemental Figures S1–S4), including 20 carbonyl compounds, 16 hydrocarbons, 8 alcohols, seven organohalogens, three terpenoids, two furanic compounds, and three others. All the VOCs identified in the samples studied are listed in Table 1, as well as the retention time (RT), KIs, and mean relative area ± standard deviation. Supplemental Table S1 reports the relative concentration of VOCs identified in the marine macroalgae by HS-SPME/GC-MS. The volatilomic fingerprint of the marine macroalgae was considerably different, since only three VOCs were common to all samples, namely hexanal, benzaldehyde and β-ionone. The contribution of each chemical family to the total volatilomic fingerprint of each marine macroalgae, is shown in Figure 5.

The carbonyl compounds were the predominant chemical family across all samples, except for the *A. taxiformis* algae (29% of total volatilomic fingerprint). Within the carbonyl family, each sample exhibited a distinct predominant VOC, for the *C. webbiana,* it was 3-cyclohepten-1-one (contributing 18.5% of the carbonyl compounds fraction), acetone in *A. taxiformis* (29.6%), hexanal in *R. okamurae* (21.1%), and for the CCA, benzaldehyde (27.6%). Interestingly, benzaldehyde was present in all four marine macroalgae in moderate amounts. This compound is characterized by a bitter almond-like aroma [24] and has been widely used as a flavoring agent [25] (Table 2). Hexanal was detected in all samples, exhibiting a substantially higher abundance in *R. okamurae* than the other algae studied, accounting for 11.5% of the total volatilomic fingerprint. This compound has been described to have a grassy, fatty odor [26], being commonly used as a fragrance ingredient in the cosmetic industry, as well as a preservative to extend the shelf life [27]. (*E*,*E*)-2,4-decadienal, (*Z*,Z)-2,4-decadienal and 2,4-heptadienal originated from the lipid degradation [28] and presented a fatty fried-like smell [24], where 2,4-heptadienal had a rancid, fishy smell [28]. These aldehydes have also been reported to possess antimicrobial activity [29]. 1-Octen-3-one was characterized by giving the algae a mushroom- and metallic-like smell, which was pronounced in the *C. webbiana* algae. 6-Methyl-5-hepten-2-one was the second most abundant carbonyl detected exclusively in *R. okamurae*. This VOC has been defined by a fruity, earthy smell [24], being used as a fragrance ingredient and possesses anti-bacterial activity [30]. This ketone is likely derived from the fatty acid oxidation [28].

The hydrocarbons are predominantly characterized by saturated chained hydrocarbons (8 out of 16), a few unsaturated (4 out of 16) and aromatic (4 out of 16), where *R. okamurae* exhibited the highest contribution to the total volatilomic fingerprint (30%), followed by coralline algae (29%), *C. webbiana* (12%) and *A. taxiformis* (7%). Pentadecane was the main VOC in *R. okamurae*, accounting for 13.9% of the total volatilomic fingerprint of this alga. Practically odorless, it is frequently applied in skin-care products. The other macroalgae had unique VOCs identified exclusively in them, with lower abundances.

*C. webbiana* and *A. taxiformis* are predominantly composed of organohalogens (Figure 5), halogenated VOCs that function as defense metabolites against herbivores, fouling organisms and microbes [9]. Previous studies have shown that these halogenated VOCs are present on algae, mainly present as brominated and chlorinated compounds [9]. Among this chemical family, bromine containing VOCs contributed most to the volatilomic fingerprint, with tribromomethane representing 19.7% and 12.7% of the total volatilomic fingerprint of *C. webbiana* and *A. taxiformis*, respectively. These halogenated VOCs present antifungal and antibacterial properties [40], and are extensively employed in various industrial sectors, particularly as pesticides, insecticides, and in pharmaceutical applications [41,42]. On the other hand, only one organohalogen VOC was detected in coralline algae, namely 2-iodopentane, which represents 2.7% of the total volatilomic fingerprint.

The terpenoids identified in the algae were β-ionone, β-cyclocitral, and geranylacetone, which resulted from carotenoid-cleaving-like enzymes (carotenoid degradation). This chemical family exhibited one of the lowest abundances amongst other chemical families, ranging from 0.8 to 3.5. The coralline algae displayed the highest β-ionone relative area (3.5). This VOC is reported to have anti-inflammatory and anticancer traits [42], with a violet-like aroma and the characteristic seaweed odor [24]. β-cyclocitral was only identified in 2 of the 4 samples (*R. okamurae* and the coralline algae) and is associated with a fresh scent [43]; in addition, it acts as an oxidative-stress protector [44]. Geranylacetone was identified only in the coralline algae, displaying a floral, fruity odor [45] and has been documented to have anti-radical activity [46]. These three VOCs are frequently employed in the cosmetic industry as fragrance ingredients [47].

The contribution of the alcohol chemical family to the total volatilomic fingerprint, on average, was lower than 5%. *A. taxiformis* only had a single alcohol detected, tridecanol, which represents 1.31% of the total volatilomic fingerprint, and is widely employed as a key ingredient in cosmetic formulations [48]. A few of these alcohols, such as 1-hexanol, were generated through the peroxidation of the unsaturated fatty acids [28]. 1-Hexanol is noted by a grassy odor [24], and is regularly applied in fragrances [49].

The furanic compounds were detected exclusively in *R. okamurae* and are likely produced through sugar dehydration or Maillard reaction fragmentation. These compounds exhibit a metallic, vegetable-like odor [28], and contribute minimally to the total volatilomic profile (2%). Dimethyl sulfide is a breakdown product of dimethylsulfoniopropionate, a tertiary sulfonium compound accumulated at high levels mainly by calcified marine algae. This sulphide compound, characterized by an eggy, onion-like odor [28] is characteristic of coralline algae [12] and accounted for 18.9% of their total volatilomic fingerprint.

### 3.3. Statistical Profiling of the Macroalgae Volatilomic Fingerprint

**Figure 6:**
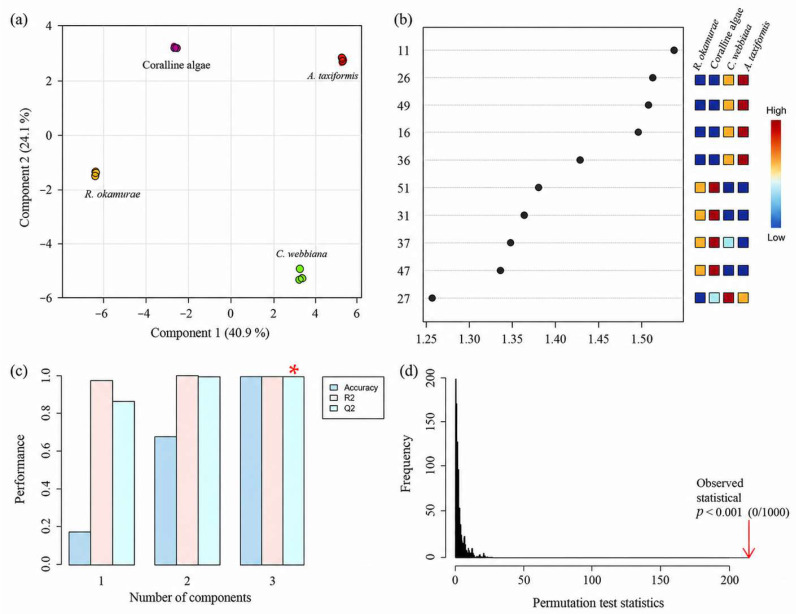
PLS-DA of the volatilomic fingerprint of the samples (*n* = 3 for each data point): (**a**) score scatter plot; (**b**) VIP scores of key features identified by PLS-DA, with colored boxes indicating relative VOCs content across groups; (**c**) 5-fold cross-validation of PLS-DA performance using varying component numbers (* denotes best Q^2^ value) and (**d**) PLS-DA model validated by 1000 permutation tests of GC-MS-derived VOCs from the marine algae investigated. For peak identification, consult Table 1.

The PLS-DA scatter plot (Figure 6a) revealed that the marine macroalgae were distinctly separated along the PC1 and PC2 principal components, accounting for 64.9% of the total variance. This separation is supported by univariate statistical analysis, which demonstrated that all 59 identified VOCs exhibited statistically significant differences.

**Figure 7:**
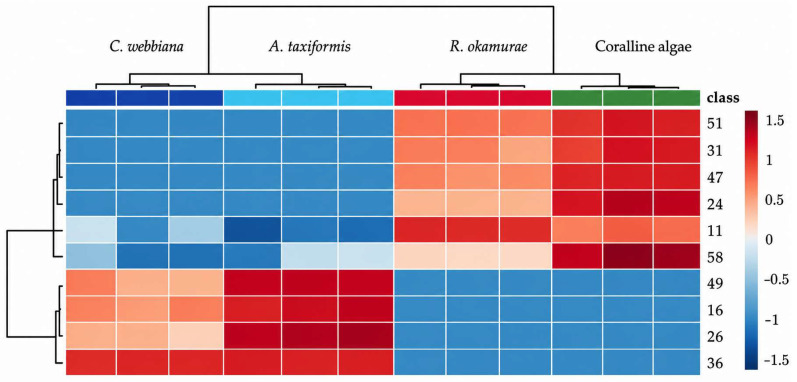
Heatmap of the putative characteristic molecular biomarkers (VIP > 1) identified in marine macroalgae. For peak identification, consult Table 1.

Figure 6b showed the ten differently expressed VOCs with VIP scores higher than 1, being 1-octen-3-one (#27), β-cyclocitral (#47), 2,4-heptadienal (#37), 1-hexanol (#31), (*E*,*E*)-2,4-decadienal (#51), tribromomethane (#36), dibromomethane (#16), hexadecane (#49), bromochloronitromethane (#26) and hexanal (#11), emerged as the most discriminative biomarkers among the algae studied, highlighting their potential as characteristic molecular markers. As evidenced earlier, the organohalogens revealed a strong correlation with both *C. webbiana* and *A. taxiformis*, with a markedly higher association observed for *A. taxiformis* relative to *C. webbiana*, as shown in Figure 6b and Figure 7. This suggests that these VOCs played a key role in shaping the volatilomic fingerprint of the red species, as it was reported that among marine red algae species, they exhibit the highest abundance of biosynthetic pathways to produce organohalogens [50]. The permutation test (Figure 6d) confirmed the statistical significance of the model (*p* < 0.001), indicating that the observed separation is unlikely to be random. The observed statistic (red arrow) falls well outside the distribution of the permuted values, supporting the robustness of the model. The model performance improved with the increasing number of components, where the three-component model achieved the highest accuracy, R^2^, and Q^2^ values, in this way indicating an excellent classification and predictive performance.

The HCA (Figure 7) resulting dendrogram provided a clear visual representation of the dataset. *R. okamurae* and the coralline algae formed distinct clusters characterized by the enrichment of specific VOCs, whereas *C. webbiana* and *A. taxiformis* exhibited more similar volatilomic fingerprints.

## 4. Conclusions

This study demonstrated that HS-SPME/GC-MS, combined with multivariate statistical analyses, provides a robust framework for characterizing and differentiating marine macroalgae based on their volatilomic fingerprint. A total of 59 VOCs were identified, revealing pronounced species-specific differences, as only three VOCs (hexanal, benzaldehyde, and β-ionone) were common to all samples. Carbonyl compounds dominated the volatilomic fingerprint of most algae, while organohalogens were particularly characteristic of *C. webbiana* and *A. taxiformis*, underscoring their ecological role in chemical defense and their relevance as discriminative molecular markers. Each alga exhibited a distinct volatilomic fingerprint shaped by key compounds: tribromomethane and 3-cyclohepten-1-one in *C. webbiana*, tribromomethane and bromochloronitromethane in *A. taxiformis*, hexanal and pentadecane in *R. okamurae*, and dimethyl sulfide and β-ionone in coralline algae. Several of these VOCs are important contributors to aroma, and are also associated with documented bioactivities, including antimicrobial, antioxidant, anti-inflammatory, and potential anticancer properties, highlighting their valorization potential beyond sensory attributes, particularly in cosmetic, nutraceutical, and pharmaceutical contexts.

Multivariate analyses clearly discriminated the algal species, demonstrating the robustness of the volatilomic dataset. Organohalogens emerged as a key chemical family for differentiating C. *webbiana* and *A. taxiformis*, while *R. okamurae* and coralline algae displayed distinct clusters supported by unique and statistically significant VOCs. The high R^2^ and Q^2^ values, together with permutation testing, confirmed the strong predictive performance and statistical validity of the models, supporting the use of these VOCs as putative biomarkers for species differentiation.

*R. okamurae* emerged as the species with the greatest bioactive potential, exhibiting the highest TPC (144 mgGAE/100 g DW), TFC (69 ± 6 mg QE/100 g DW), and antioxidant capacity across both assays, compared to *A. taxiformis*, *C. webbiana* and coralline algae. These findings provide a framework for valorizing invasive or less utilized macroalgal biomass for bioproduct development, a strategy increasingly supported by the current technological advances in algal biology, extraction, and bioproduct development. Further studies should investigate the effects of cultivation and processing of the biomass on VOC yields and composition, as well as the techno-economic viability of scaling up volatile extraction to fully exploit the potential of macroalgae within a sustainable bioeconomy. This study should be regarded only as a preliminary investigation with the intent to valorize the cosmetic and industrial applications of the selected algae. Therefore, the results presented here do not represent the definitive values of these species, but rather provide an initial characterization to support future works.

## Figures and Tables

**Table 1:** Relative area ± standard deviation of volatile organic compounds identified in the marine macroalgae by HS-SPME/GC-MS.

Peak nº	RT (min)	KI_calc_	KI_lit_	Chemical Families	*C.* *webbiana*	*A.* *taxiformis*	*R.* *okamurae*	Coralline Algae
				**Carbonyl compounds**				
2	9.76	820	821	Acetone	1.68 ± 0.01	3.3 ± 0.6	2.7 ± 0.4	-
3	13.5	845	826	3-Cyclohepten-1-one	4.7 ± 0.4	-	-	-
6	15.3	991	973	1-Pente-3-one	-	-	2.2 ± 0.3	-
11	17.4	1056	1034	Hexanal	1.8 ± 0.3	0.6 ± 0.1	9.3 ± 1.5	2.80 ± 0.01
13	19.1	1103	1101	2-Methyl-2-butenal	-	-	3.0 ± 0.6	-
21	22.3	1186	1187	(*Z*)-2-hexenal	-	-	3.1 ± 0.3	-
22	23.7	1222	1218	3-octanone	3.1 ± 0.6	0.19 ± 0.03	0.68 ± 0.08	-
24	24.9	1253	1248	Octanal	-	-	0.37 ± 0.03	0.8 ± 0.1
27	25.8	1273	1277	1-Octen-3-one	4.6 ± 0.4	0.9 ± 0.1	-	0.50 ± 0.01
30	26.8	1299	1314	6-Methyl-5-hepten-2-one	-	-	7.5 ± 1.2	-
32	29.1	1368	1367	Nonanal	0.77 ± 0.06	-	1.2 ± 0.1	0.91 ± 0.02
34	30.2	1397	1392	(*E*)-2-octenal	1.0 ± 0.1	-	1.24 ± 0.03	1.01 ± 0.03
37	32.4	1466	1458	2,4-Heptadienal	0.46 ± 0.04	-	1.3 ± 0.2	0.82 ± 0.03
38	32.7	1475	1497	Decanal	0.9 ± 0.1	-	-	1.31 ± 0.02
40	33.4	1497	1482	Benzaldehyde	2.9 ± 0.4	2.3 ± 0.4	4.1 ± 0.7	3.4 ± 0.3
48	39.2	1622	1624	3-Methylbenzaldehyde	-	0.48 ± 0.06	-	-
51	40.5	1732	1739	(*E*,*E*)-2,4-decadienal	-	-	1.2 ± 0.2	0.8 ± 0.1
54	41.6	1843	1824	(*Z*,*Z*)-2,4-decadienal	0.8 ± 0.1	0.56 ± 0.07	1.2 ± 0.2	-
56	42.2	1945	1940	Tetradecanal	1.8 ± 0.3	1.4 ± 0.2	4.2 ± 0.4	-
59	47.9	2085	2060	Hexadecanal	0.95 ± 0.04	1.4 ± 0.1	1.02 ± 0.12	-
				**Hydrocarbons**				
4	15.0	980	1000	Decane	-	-	-	1.71 ± 0.02
8	15.9	1010	997	3,5-Dimethyl-1,6-octadiene	-	-	1.2 ± 0.2	-
9	16.1	1016	1023	2,2,4,4-Tetramethyloctane	0.45 ± 0.09	-	-	-
12	18.0	1073	1082	2-Ethyl-1,3-dimethylbenzene	0.9 ± 0.1	-	3.8 ± 0.6	-
17	20.8	1148	1144	1,2,3,4-Tetramethylbenzene	0.52 ± 0.07	-	-	-
18	21.3	1162	1186	1,3,5,8-Undecatetraene	0.7 ± 0.1	-	-	-
20	22.2	1183	1200	Dodecane	1.3 ± 0.2	-	-	1.9 ± 0.2
29	26.4	1292	1300	Tridecane	-	-	-	0.39 ± 0.02
33	29.9	1390	1400	Tetradecane	1.3 ± 0.2			0.78 ± 0.01
39	33.0	1485	1500	Pentadecane	-	-	11 ± 1	-
44	34.3	1527	1513	(*Z*)-5-Pentadecene	-	-	2.6 ± 0.5	-
41	33.7	1507	1525	1,2,3,4-Tetrahydro-naphthalene	-	-	2.0 ± 0.2	-
46	35.5	1567	1582	6-Methyl heptadiene	-	-	2.5 ± 0.1	-
49	39.5	1624	1600	Hexadecane	0.6 ± 0.1	2.2 ± 0.3	-	-
50	40.2	1730	1715	Naphthalene	1.6 ± 0.1	0.64 ± 0.04	1.2 ± 0.3	
52	40.7	1745	1754	3-Methyl-heptadecane	-	-	-	1.2 ± 0.1
				**Alcohols**				
7	15.7	1003	1005	2-Methyl-3-buten-2-ol	-	-	0.77 ± 0.08	-
28	26.3	1288	1288	(*Z*)-2-penten-1-ol	-	-	1.1 ± 0.2	-
35	30.3	1404	1406	1-Octen-3-ol	-	-	-	0.68 ± 0.02
31	27.4	1316	1308	1-Hexanol	-	-	0.8 ± 0.2	0.65 ± 0.04
43	33.9	1514	1519	1-Octanol	-	-	0.6 ± 0.1	-
45	34.7	1540	1550	Decan-5-ol	0.7 ± 0.1	-	-	-
53	41.2	1838	1832	9-Decen-1-ol	0.6 ± 0.1	-	-	-
57	42.3	1946	1952	Tridecanol	2.2 ± 0.3	0.5 ± 0.1	2.5 ± 0.2	-
				**Organohalogens**				
5	15.1	983	-	1,2-Dibromoethene	-	0.6 ± 0.1	-	-
10	16.3	1023	1036	1-Bromoheptane	-	0.8 ± 0.1	-	-
14	19.5	1114	1101	Bromotrichloromethane	-	2.3 ± 0.4	-	-
15	19.7	1116	1114	2-Iodopentane	-	-	-	0.87 ± 0.04
16	19.8	1122	1136	Dibromomethane	0.8 ± 0.1	2.1 ± 0.3	-	-
26	25.3	1263	-	Bromochloronitromethane	0.4 ± 0.1	4.04 ± 0.22	-	-
36	30.5	1408	1407	Tribromomethane	19 ± 3	13 ± 2	-	-
				**Terpenoids**				
47	36.6	1601	1584	β-Cyclocitral	-	-	0.9 ± 0.2	0.9 ± 0.1
55	42.0	1896	1883	Geranylacetone	-	-	-	1.2 ± 0.1
58	43.3	1953	1958	β-ionone	1.1 ± 0.1	0.8 ± 0.1	3.1 ± 0.6	3.5 ± 0.3
				**Furanic compounds**				
19	22.1	1181	1198	2-Pentyl furan	-	-	1.4 ± 0.1	-
25	25.0	1254	1262	(*E*)-2-(2-Pentenyl)furan	-	-	0.35 ± 0.02	-
				**Others**				
1	8.73	735	716	Dimethyl sulfide	-	-	-	6.1 ± 0.8
23	24.0	1231	1251	Hexyl acetate	-	-	0.37 ± 0.05	-
42	33.9	1512	-	3-Ethyl phenol	5.7 ± 0.5	-	-	-

RT—retention time; KI_calc_—calculated Kovat index relative n-alkanes (C8 to C20) on a SUPELCOWAX^®^ 10 capillary column; KI_lit_—literature Kovat index; -: not detected.

**Table 2:** Predominant VOCs with documented bioactive activities detected in the studied marine macroalgae.

VOCs	*C.* *webbiana*	*A.* *taxiformis*	*R.* *okamurae*	Coralline Algae	Bioactive Properties	Ref.
Hexanal	*x*	x	x	x	Antimicrobial, natural preservative	[31]
Benzaldehyde	*x*	x	x	x	Antimicrobial, insecticidal	[32]
6-Methyl-5-hepten-2-one			x		Vasorelaxant effect, antioxidant	[33]
Pentadecane			x		Anti-inflammatory, analgesic	[34]
Tribromomethane	x	x			Antimethanogenic, antimicrobial	[35]
Bromochloronitromethane	*x*	x			-	-
β-Ionone	x	x	x	x	Anticancer, antioxidant	[36]
Dimethyl Sulfide				x	Antibacterial, antioxidant	[37,38]
3-Ethylphenol	x				Antioxidant	[39]

## Data Availability

The original contributions of this study are fully included in the article. Any additional inquiries should be addressed to the corresponding authors.

## Supplementary Materials

The following supporting information can be downloaded at https://www.mdpi.com/article/10.3390/biology15141129/s1, Figure S1: Typical chromatogram of *Caulerpa webbiana* obtained using HS-SPME/GC-MS; Figure S2: Typical chromatogram of *Asparagopsis taxiformis* obtained using HS-SPME/GC-MS; Figure S3: Typical chromatogram of *Rugulopteryx okamurae* obtained using HS-SPME/GC-MS; Figure S4: Typical chromatogram of the coralline algae obtained using HS-SPME/GC-MS; Table S1: Relative concentration (µg/L) ± standard deviation of volatile organic compounds identified in the marine macroalgae by HS-SPME/GC-MS.

## Abbreviations

The following abbreviations are used in this manuscript:

HS-SPME/GC-MS	headspace solid-phase microextraction tandem gas chromatography–mass spectrometry
HS-SPME	headspace solid-phase microextraction
VOCs	Volatile organic compounds
TPC	Total phenolic content
TFC	Total flavonoid content
DPPH	2,2-diphenyl-1-picrylhydrazyl
ABTS	2,2′-azino-bis(3-ethylbenzothiazoline-6-sulfonic acid)
CCA	Crustose coralline algae
DVB/CAR/PDMS	divinylbenzene/carboxen/polydimethylsiloxane
PTFE	Polytetrafluoroethylene
DMSP	Dimethylsulphoniopropionate
DMS	Dimethyl sulphide
SD	Standard deviation
*C. webbiana*	*Caulerpa webbiana*
*A. Taxiformis*	*Asparagopsis taxiformis*
*R. okamurae*	*Rugulopteryx okamurae*
mgGAE/100 g DW	milligrams of gallic acid equivalents per 100 g of dry weight
mgQE/100 g DW	milligrams of quercetin equivalents per 100 g of dry weight
mgTE/100 g DW	milligrams of trolox equivalents per 100 g of dry weight
PLS-DA	Partial Least Squares–Discriminant Analysis
HCA	Hierarchical Cluster Analysis
PC1	Principal component 1
PC2	Principal component 2
RT	Retention time
KI_calc_	calculated Kovat index relative n-alkanes
kI_lit_	literature Kovat index
VIP	Variable Importance in Projection
