# Supplementary material for: Discriminating Marine Macroalgae by Volatilomic Fingerprint and Bioactivity: A Chemometric Approach
Source: Biology (Basel). 2026 Jul 11;15(14):1129. doi: 10.3390/biology15141129 (PMC13406038; doi:10.3390/biology15141129)
Supplement: Supplementary file 1 [file biology-15-01129-s001.zip › biology-4372476-supplementary.pdf]

*Article*

# Discriminating Marine Macroalgae by Volatilomic Fingerprint and Bioactivity: A Chemometric Approach

**Gonçalo Jasmins [1], Rosa Perestrelo [1], Ricardo Luís [2], Rodrigo Silva [2], Pedro Sousa [2], Carlos A. P. Andrade [2*], José Câmara [1,3*]**

[1] CQM - Centro de Química da Madeira, Universidade da Madeira, Campus da Penteada, 9020-105 Funchal, Portugal. goncalo.jasmins@staff.uma.pt; rmp@staff.uma.pt

[2] MARE – Marine and Environmental Sciences Centre / ARNET – Aquatic Research Network, Agência Regional Para o Desenvolvimento da Investigação Tecnologia e Inovação (ARDITI), Funchal, Madeira, Portugal. pedro.sousa@mare.arditi.pt; rodrigo.silva@mare.arditi.pt; ricardo.luis@arditi.pt; carlos.andrade@mare.arditi.pt

[3] Departamento de Química, Faculdade de Ciências Exatas e Engenharia, Universidade da Madeira, Campus da Penteada, 9020-105 Funchal, Portugal. jsc@staff.uma.pt

* Correspondence: carlos.andrade@mare.arditi.pt; jsc@staff.uma.pt

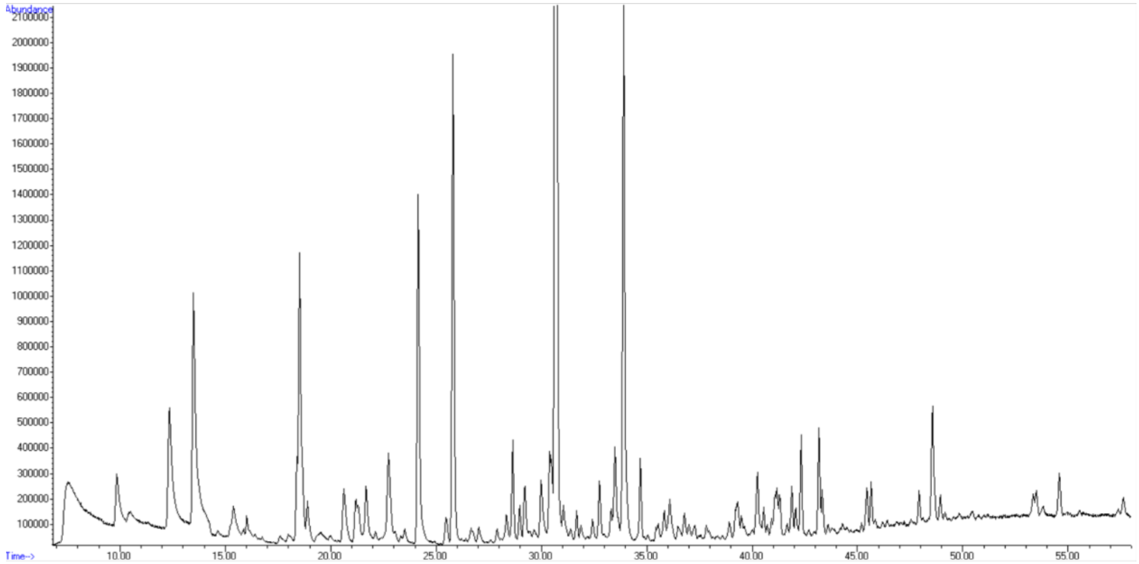


Figure S1: Typical chromatogram of *Caulerpa webbiana* obtained using HS-SPME/GC-MS

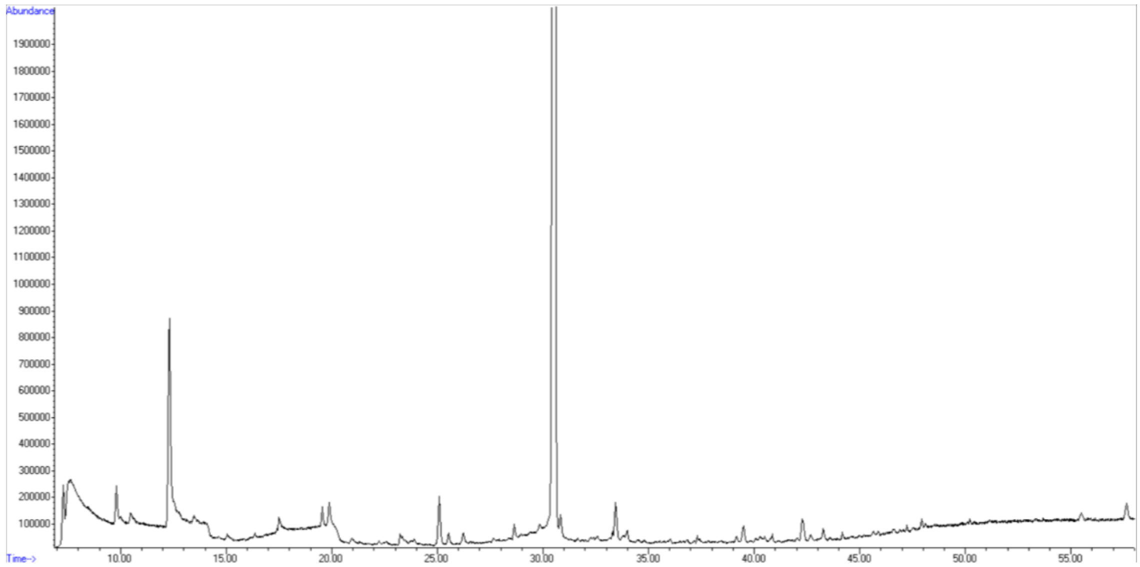


Figure S2: Typical chromatogram of *Asparagopsis taxiformis* obtained using HS-SPME/GC-MS

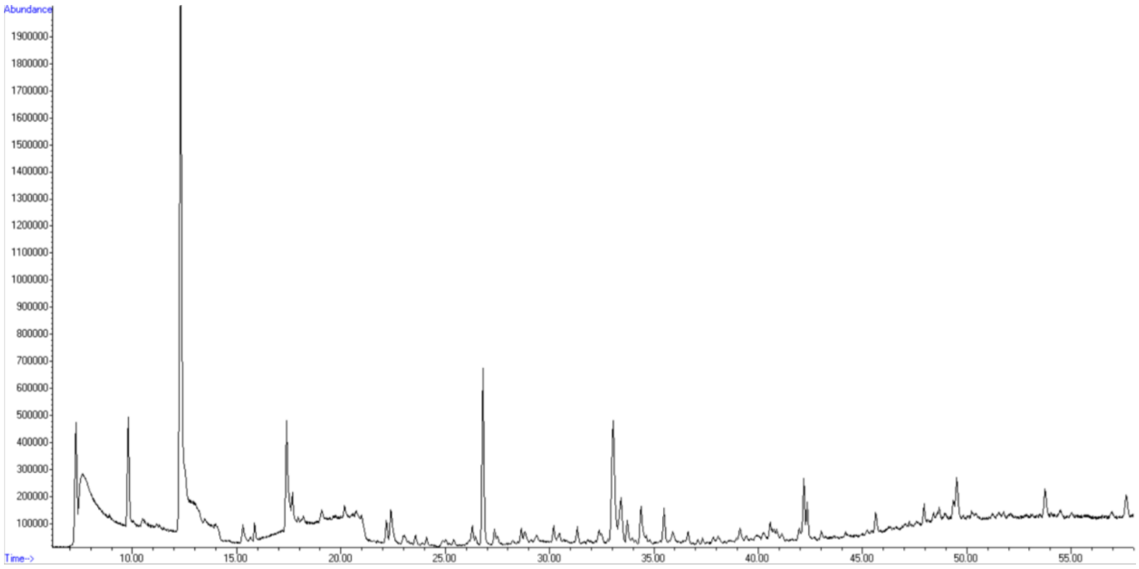


Figure S3: Typical chromatogram of *Rugulopteryx okamurae* obtained using HS-SPME/GC-MS

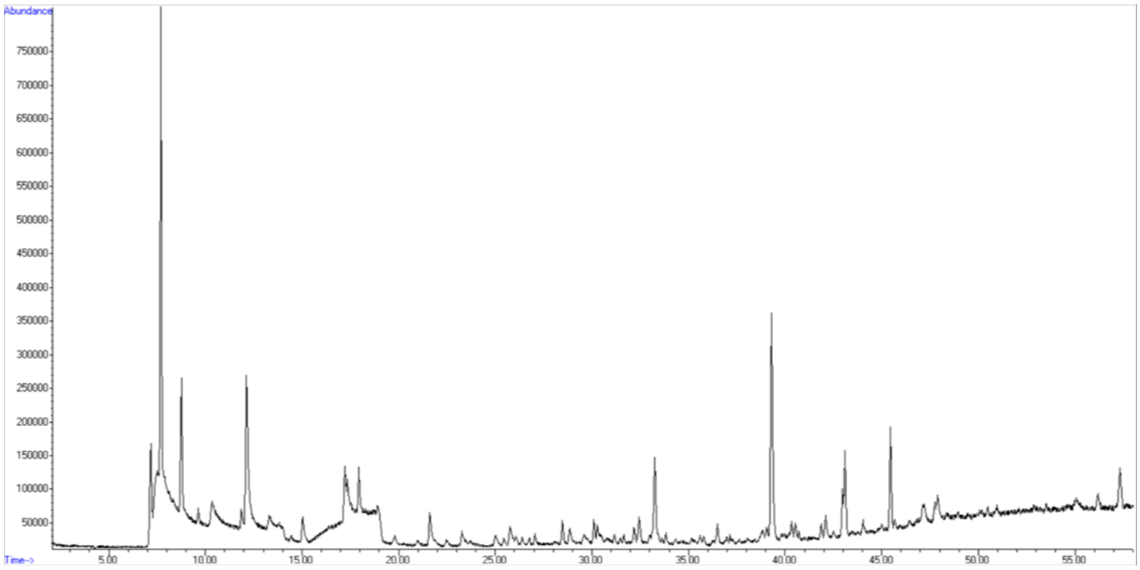


Figure S4: Typical chromatogram of the coralline algae obtained using HS-SPME/GC-MS

Table S1. Relative concentration (μg/L) ± standard deviation of volatile organic compounds identified in the marine macroalgae by HS-SPME/GC-MS.

| **RT (min)** | **VOCs** | ***C. webbiana*** | ***A. taxiformis*** | ***R. okamurae*** | **Coralline algae** |
|---|---|---|---|---|---|
| 8.73 | Dimethyl sulfide | - | - | - | 101 ± 17 |
| 9.76 | Acetone | 27.5 ± 0.3 | 55 ± 12 | 43 ± 8 | - |
| 13.47 | 3-Cyclohepten-1-one | 77 ± 8 | - | - | - |
| 15.03 | Decane | - | - | - | 28.2 ± 0.3 |
| 15.05 | 1,2-Dibromoethene | - | 10 ± 2 | - | - |
| 15.3 | 1-Pente-3-one | - | - | 36 ± 6 | - |
| 15.66 | 2 -Methyl-3-buten-2-ol | - | - | 12 ± 2 | - |
| 15.86 | 3,5-Dimethyl-1,6-octadiene | - | - | 20 ± 5 | - |
| 16.06 | 2,2,4,4-Tetramethyloctane | 7 ± 2 | - | - | - |
| 16.30 | 1-bromoheptane | - | 12 ± 3 | - | - |
| 17.39 | Hexanal | 29 ± 6 | 9.63 ± 1.48 | 153 ± 29 | 46.2 ± 0.3 |
| 17.98 | 2-Ethyl-1,3-dimethylbenzene | 15 ± 3 | - | 63 ± 12 | - |
| 19.09 | 2-Methyl-2-butenal | - | - | 48 ± 11 | - |
| 19.50 | Bromotrichloromethane | - | 38 ± 7 | - | - |
| 19.70 | 2-Iodopentane | - | - | - | 14.2 ± 0.7 |
| 19.80 | Dibromomethane | 14 ± 2 | 34 ± 6 | - | - |
| 20.75 | 1,2,3,4-Tetramethylbenzene | 8 ± 1 | - | - | - |
| 21.30 | 1,3,5,8-Undecatetraene | 12 ± 1 | - | - | - |
| 22.09 | 2-Pentyl furan | - | - | 22 ± 3 | - |
| 22.17 | Dodecane | 21 ± 4 | - | - | 32 ± 4 |
| 22.31 | (Z)-2-hexenal | - | - | 51 ± 6 | - |
| 23.72 | 3-octanone | 51 ± 12 | 3.1 ± 0.6 | 11 ± 2 | - |
| 24.03 | Hexyl acetate | - | - | 6 ± 1 | - |
| 24.88 | Octanal | - | - | 6.2 ± 0.7 | 17 ± 1 |
| 24.95 | (E)-2-(2-Pentenyl)furan | - | - | 5.7 ± 0.4 | - |
| 25.33 | Bromochloronitromethane | 7 ± 1 | 66 ± 4 | - | - |
| 25.78 | 1-Octen-3-one | 75 ± 8 | 15 ± 3 | - | 8.4 ± 0.2 |
| 26.3 | (Z)-2-penten-1-ol | - | - | 18 ± 4 | - |
| 26.42 | Tridecane | - | - | - | 6.9 ± 0.4 |
| 26.77 | 6-Methyl-5-hepten-2-one | - | - | 122 ± 25 | - |
| 27.35 | 1-hexanol | - | - | 13 ± 3 | 9.3 ± 0.9 |
| 29.10 | Nonanal | 13 ± 1 | - | 19 ± 2 | 14.3 ± 0.4 |
| 29.89 | Tetradecane | 22 ± 5 | - | - | 13.1 ± 0.3 |
| 30.15 | (E)-2-octenal | 17 ± 2 | - | 20.3 ± 0.6 | 16.3 ± 0.7 |
| 30.28 | 1-Octen-3-ol | - | - | - | 11.5 ± 0.5 |
| 30.50 | Tribromomethane | 316 ± 68 | 208 ± 45 | - | - |
| 32.35 | 2,4-Heptadienal | 7.5 ± 0.8 | - | 22 ± 5 | 13.1 ± 0.6 |
| 32.67 | Decanal | 15 ± 3 | - | - | 20.5 ± 0.3 |
| 32.98 | Pentadecane | - | - | 183 ± 25 | - |

| | | | | | |
|---|---|---|---|---|---|
| 33.40 | Benzaldehyde | 47 ± 8 | 38 ± 7 | 67 ± 14 | 55 ± 6 |
| 33.70 | 1,2,3,4-Tetrahydro-naphthalene | - | - | 33 ± 5 | - |
| 33.85 | 3-Ethyl phenol | 93 ± 9 | - | - | - |
| 33.92 | 1-Octanol | - | - | 10 ± 2 | - |
| 34.34 | (Z)-5-Pentadecene | - | - | 42 ± 10 | - |
| 34.67 | Decan-5-ol | 12 ± 2 | - | - | - |
| 35.48 | 6-Methyl heptadiene | - | - | 41 ± 2 | - |
| 36.63 | β-Cyclocitral | - | - | 15 ± 3 | 15 ± 1 |
| 39.15 | 3-Methylbenzaldehyde | - | 8 ± 1 | - | - |
| 39.48 | Hexadecane | 9 ± 2 | 36 ± 6 | - | - |
| 40.21 | Naphthalene | 26 ± 2 | 10.5 ± 0.7 | 20 ± 6 | - |
| 40.53 | (E,E)-2,4-decadienal | - | - | 20 ± 3 | 13 ± 2 |
| 40.65 | 3-Methyl-heptadecane | - | - | - | 16 ± 1 |
| 41.17 | 9-Decen-1-ol | 10 ± 2 | - | - | - |
| 41.61 | (Z,Z)-2,4-decadienal | 13 ± 3 | 9 ± 1 | 19 ± 4 | - |
| 41.97 | Geranylacetone | - | - | - | 20 ± 2 |
| 42.15 | Tetradecanal | 29 ± 6 | 23 ± 4 | 67 ± 9 | - |
| 42.30 | Tridecanol | 35 ± 6 | 9 ± 2 | 41 ± 4 | - |
| 43.27 | β-ionone | 17 ± 3 | 13 ± 2 | 50 ± 12 | 57 ± 6 |
| 47.93 | Hexadecanal | 15.6 ± 0.8 | 22 ± 3 | 17 ± 2 | - |
